# Identification of a novel *PRUNE2::NTRK2* gene fusion in soft tissue sarcoma patients—friend or foe? Case series

**DOI:** 10.1177/17588359251395379

**Published:** 2025-11-27

**Authors:** Klaudia Bobak, Andrzej Tysarowski, Katarzyna A. Seliga, Jakub Pia̧tkowski, Paweł Golik, Mateusz J. Spałek, Anna Szumera-Ciećkiewicz, Piotr Rutkowski, Anna M. Czarnecka

**Affiliations:** Department of Soft Tissue/Bone Sarcoma and Melanoma, Maria Sklodowska-Curie National Research Institute of Oncology, W. K. Roentgena 5, Warsaw 02-781, Poland; Cancer Molecular and Genetic Diagnostics Laboratory, Maria Sklodowska-Curie National Research Institute of Oncology, Warsaw, Poland; Cancer Molecular and Genetic Diagnostics Laboratory, Maria Sklodowska-Curie National Research Institute of Oncology, Warsaw, Poland; Faculty of Biology, Institute of Genetics and Biotechnology, University of Warsaw, Warsaw, Poland; Faculty of Biology, Institute of Genetics and Biotechnology, University of Warsaw, Warsaw, Poland; Department of Soft Tissue/Bone Sarcoma and Melanoma, Maria Sklodowska-Curie National Research Institute of Oncology, Warsaw, Poland; Department of Radiotherapy I, Maria Sklodowska-Curie National Research Institute of Oncology, Warsaw, Poland; Department of Cancer Pathomorphology, Maria Sklodowska-Curie National Research Institute of Oncology, Warsaw, Poland; Diagnostic Hematology Department, Institute of Hematology and Transfusion Medicine, Warsaw, Poland; Department of Soft Tissue/Bone Sarcoma and Melanoma, Maria Sklodowska-Curie National Research Institute of Oncology, Warsaw, Poland; Department of Soft Tissue/Bone Sarcoma and Melanoma, Maria Sklodowska-Curie National Research Institute of Oncology, Warsaw, Poland; Department of Experimental Pharmacology, Mossakowski Medical Research Institute, Polish Academy of Sciences, Warsaw, Poland

**Keywords:** case report, gene fusion, novel fusion, *NTRK2*, *PRUNE2*, soft tissue sarcoma

## Abstract

Soft tissue sarcomas (STS) are rare mesenchymal tumors in which gene fusions occur in approximately one-third of cases, serving as key diagnostic and therapeutic targets. This study investigates the presence and implications of gene fusions in STS, focusing on a novel *PRUNE2::NTRK2* gene fusion identified in two adult patients. The *PRUNE2* gene plays a role in cellular processes and is a potential tumor biomarker. PRUNE2 plays a role in various tumors as a tumor suppressor, including prostate cancer, colorectal cancer, and neuroblastoma. The *NTRK2* oncogene is, however, associated with tumor progression. In this report, we describe a possible molecular characterization of a novel *PRUNE2::NTRK2* gene fusion. Although *NTRK*-associated fusions are significant in various cancers and have led to the development of targeted therapies, such as larotrectinib and entrectinib, the specific molecular impact of atypical *PRUNE2::NTRK2* fusion remains unclear. The *PRUNE2::NTRK2* gene fusions described here express a non-functional TrkB protein, and it is unclear whether the PRUNE2 function is intact or affected.

## Introduction

Soft tissue sarcomas (STS) are a heterogeneous group of rare mesenchymal neoplasms.^1^ In approximately one-third of STS, gene fusions are prevalent. Gene fusions are important driver mutations in STS and have therefore emerged as potential treatment targets.^2^ Understanding these fusions has advanced tumor classification and patient management, especially in STS, which comprises more than 70 morphological entities.^3,4^ Even with extensive immunohistochemical markers, genetic analyses for specific fusions are crucial, especially in rare cases or when differential diagnosis is challenging.^2^ The most frequent gene fusions are the *SS18-SSX* family specific for synovial sarcomas,^2,4,5^ *FUS::DDIT3* specific for myxoid liposarcomas,^2,4,6–8^ *COL1A1::PDGFB* specific for dermatofibrosarcomas protuberans,^2,8,9^ *PAX3::FOXO1* and *PAX7::FOXO1* fusions specific for alveolar rhabdomyosarcomas,^2,4,8,10^ *NAB2::STAT6* specific for solitary fibrous tumors,^2,11,12^ and *EWSR1::FLI1* specific for Ewing sarcoma.^2,13,14^

The literature also reveals a range of case studies describing neurotrophic receptor tyrosine kinase (*NTRK*) gene fusions in STS.^15^ However, *NTRK*-associated gene fusions are rare alterations. The frequency of *NTRK*-associated gene fusion is approximately 1% of all solid tumors,^16,17^ and in STS, less than 1%.^16–18^ However, in rare cancers, the frequency of *NTRK*-associated gene fusions is up to 90%.^15,16^ For instance, in infantile fibrosarcoma, the *ETV6::NTRK3* gene fusion was reported in more than 90% of cases.^15^ In addition, *LMNA::NTRK1* and *STRN::NTRK2* gene fusions not associated with the subtype were found in some sarcoma cases.^19–23^

The tropomyosin receptor kinases (Trk), the family of receptor tyrosine kinases, TrkA, TrkB, and TrkC, are encoded by the *NTRK1*, *NTRK2*, and *NTRK3* oncogenes, respectively.^17,18^ The *NTRK1* gene is located on chromosome 1q21-q22, the *NTRK2* gene is located on chromosome 9q22.1, and the *NTRK3* gene is located on chromosome 15q25.^17^ Trk receptors have three components: an extracellular domain, a transmembrane region, and an intracellular region that contains the tyrosine kinase domain. The intracellular region contains five essential tyrosine residues, including three necessary for full kinase activity and two that act as phosphorylation-dependent binding sites for cytoplasmic adaptors and enzymes.^17,24^ Trk receptors are activated by a group of four proteins known as neurotrophins. Each neurotrophin binds to a particular Trk with high affinity. Nerve growth factor binds to TrkA, brain-derived neurotrophic factor, and neurotrophin 4 and 5 (NT-4/5) bind to TrkB, and neurotrophin 3 (NT-3) binds to TrkC.^16,17,25^ NT-3 can bind TrkA and B, but with a lower affinity.^17,24,25^ Alternative splicing can alter the interaction between a Trk receptor and its specific neurotrophin.^17^ The ligand-dependent activation of the Trk transmembrane protein initiates downstream signaling that affects cell proliferation, differentiation, and survival. Activation of TrkA initiates MAPK signaling, leading to increased cellular proliferation and growth. Active TrkB leads to activation of the MAPK, PI3K/Akt, and PLCγ pathways, resulting in neuronal differentiation and survival. Activated TrkC promotes activation of the PI3K/Akt pathway, preventing apoptosis and increasing cell survival.^16,17^ Trk signaling plays a critical role in developing the nervous system.^15,18^ Alterations of *NTRK* genes can induce carcinogenesis in neurogenic and nonneurogenic cells.^16,26^

In this study, we identify a novel *PRUNE2::NTRK2* gene fusion in leiomyosarcoma and myxofibrosarcoma patients. The human prune homolog 2 (*PRUNE2*) is identified as a susceptibility gene for Alzheimer’s disease.^27–29^ It exhibits high levels of expression in the human nervous system and brain.^28,29^ PRUNE2 plays a role in various tumors as a tumor suppressor, including prostate cancer, colorectal cancer, and neuroblastoma.^28,29^ Studies have shown its potential as a biomarker to distinguish leiomyosarcoma from gastrointestinal stromal tumors.^28,30^

## Clinical case presentation

### Case 1

**Figure 1.**
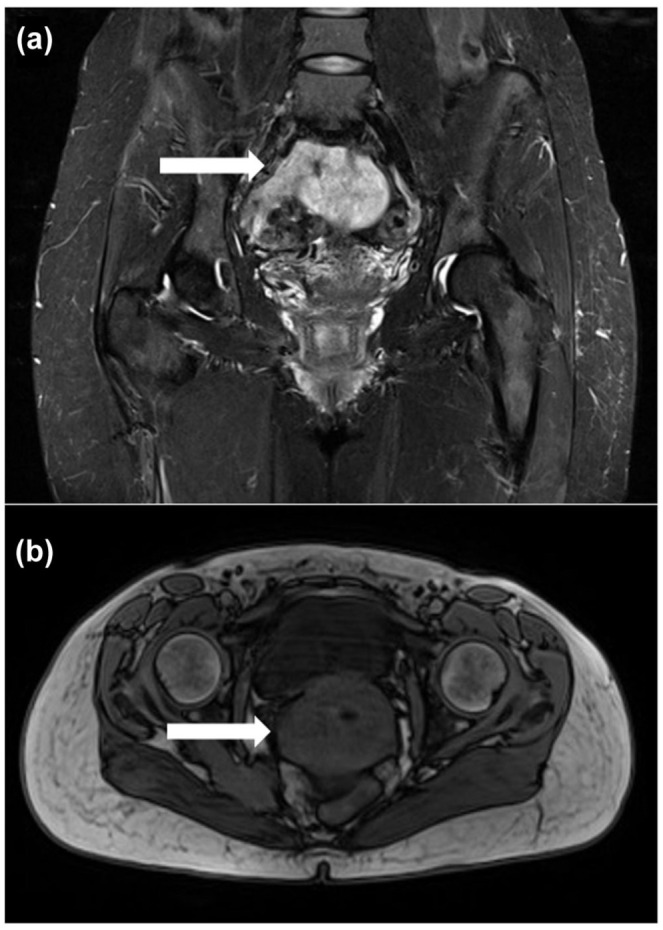
The MRI image of a pelvic mass of leiomyosarcoma on the right side: (a) contrast-enhanced coronal view and (b) axial view without contrast. MRI-magnetic resonance imaging.

In September 2017, a 41-year-old Polish woman was referred to the Oncology Clinic due to pain in the piriformis muscle. The patient had no previous chronic treatment. Ultrasound (USG) of the left buttock showed inflammatory/posttraumatic changes in the attachment of the right hip rotator muscles, 31 × 19 mm, with signs of increased perfusion and blurring of muscle structures: chronic overload tear with possible differentiation with a pathological tear. Magnetic resonance imaging (MRI) examination showed a pelvic mass on the right side of approximately 75 mm, ill-defined (Figure 1). We suspected sarcoma due to its location and qualified for biopsy by laparotomy.

**Figure 2.**
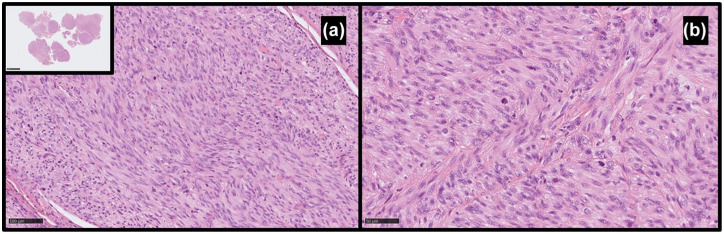
Histopathological image of the hematoxylin and eosin staining of leiomyosarcoma. (a) Spindle cell neoplasm with moderate cellular atypia (200×). (b) Fields with brisk mitotic activity (400×).

After biopsy, a histopathological examination revealed fragments of a spindle cell tumor infiltrating skeletal muscles and adipose and fibrous tissues. It was characterized by moderate atypia, mitotic activity up to 18 mitoses/10 HPF, and no necrosis. Positive immunohistochemical stainings were observed for smooth muscle actin (SMA), caldesmon, and desmin, and focal and weakly positive for CK PAN (clone AE1/AE3). Tumor cells were negative for CD34, S100, and estrogen receptor. The microscopic image with immunoprofile corresponded to the diagnosis of leiomyosarcoma grade 2 (Figure 2).

Following diagnosis, the patient was recruited for a phase II clinical trial UNRESARC (NCT03651375) conducted in our department.^31^ MRI examination before treatment showed an 82 mm tumor size. The patient was treated with preoperative chemoradiotherapy. The patient received three cycles of doxorubicin–ifosfamide (AI) according to the European Organization for Research and Treatment of Cancer–Soft Tissue and Bone Sarcoma Group (EORTC–STBSG) recommendations—ifosfamide (2500 mg/m^2^ on days 1–4) and doxorubicin (25 mg/m^2^ on days 1–3).^32^ The first AI infusion was started on December 18, 2017. One-week hypofractionated radiotherapy (a total dose of 25 Gy in 5 Gy/fraction) was started on December 28, 2017. The third AI cycle was administered on January 29, 2018. MRI examination revealed a decrease in the tumor size from 82 mm before treatment to 75 mm after treatment. The tumor was considered resectable, and surgery was performed on February 27, 2018.

On histopathological examination, the subfascial tumor had the largest dimension of 63 mm (pT2 stage). The tumor tissue stained with hematoxylin and eosin (H&E) and fibrosis areas represented 60% and 40% of the microscopically examined tumor; no necrosis was found. According to the EORTC-STBSG recommendations,^33^ the pathological response score was E grade. Sarcoma tissue was visible in the lateral and deep incision margins (resection R2). After surgery in June 2018, the patient received an adjuvant radiotherapy boost (30 Gy in 2 Gy/fraction) to the postoperative area with a margin. The patient was disease-free for more than 33 months after treatment.

In April 2021, positron emission tomography-computed tomography (PET-CT) revealed a metabolically active pathological tissue structure in the sartorius muscle in the distal part of the medial surface of the right thigh. Furthermore, the examination showed recurrence or metastasis in the surgical area in the pelvis on the right side of the ischiorectal fossa with a metabolically active pathological tissue structure at the level of the right thigh of the sciatic foramen. The patient was treated with chemotherapy with doxorubicin–dacarbazine (ADIC)–doxorubicin (12–15 mg/m^2^ on days 1–5) and dacarbazine (150–200 mg/m^2^ on days 1–5). The six cycles of ADIC infusions were administered between May and September 2021. After treatment, a PET-CT scan showed a partial regression of the disease. However, 3 months later, the next PET-CT scan showed further progression of the disease. Therefore, as a second line of treatment, the patient received 14 cycles of gemcitabine–docetaxel chemotherapy between January and July 2022.

Due to the occurrence of a new lesion and the further progression of the disease revealed in the PET-CT scan in June 2022, the treatment regimen was changed to chemotherapy with trabectedin. Between August 2022 and December 2023, the patient received 21 cycles of chemotherapy. In January 2023, a PET-CT scan showed a partial response to treatment with trabectedin (2.25 mg/m^2^). However, in May 2023, the progression of the lesion in the left lung was detected on a PET-CT scan. For this reason, in June 2023, the patient received additional radiotherapy (a total dose of 30 Gy in 6 Gy/fraction) to treat the lung lesion.

Another PET-CT scan from November 2023 showed further disease progression and local recurrence. Therefore, the patient started treatment with pazopanib (800 mg, daily) in January 2024. However, in March 2024, the dose was reduced to 600 mg daily as a result of increased transaminase levels. In August 2024, the patient was hospitalized for sepsis. During hospitalization, the patient was observed with an ulceration above the upper pole of the sacrum with intestinal contents leaking out. On August 16, 2024, a computed tomography (CT) scan revealed a significant progression of the disease (the examination was compared to 2021) and an intestinal fistula penetrating the tumor in the buttock area. The patient was surgically consulted, but surgical treatment was not possible.

In September 2024, the general condition significantly deteriorated since the previous visit. Blood tests showed a high level of C-reactive protein and creatinine, and decreased hemoglobin level. It was decided to create a stoma. As a consequence of the progression after the fourth line of chemotherapy and the inoperable nature of the disease, treatment was terminated, and additional palliative care was prescribed within the home hospice.

### Case 2

**Figure 3.**
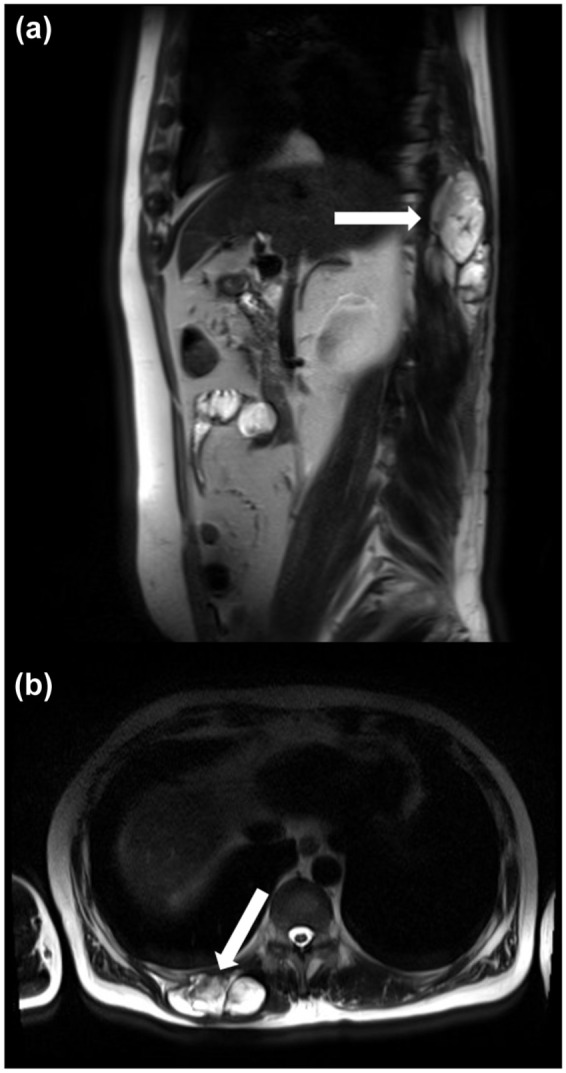
The MRI contrast-enhanced image of myxofibrosarcoma: (a) axial view and (b) sagittal view. MRI-magnetic resonance imaging.

**Figure 4.**
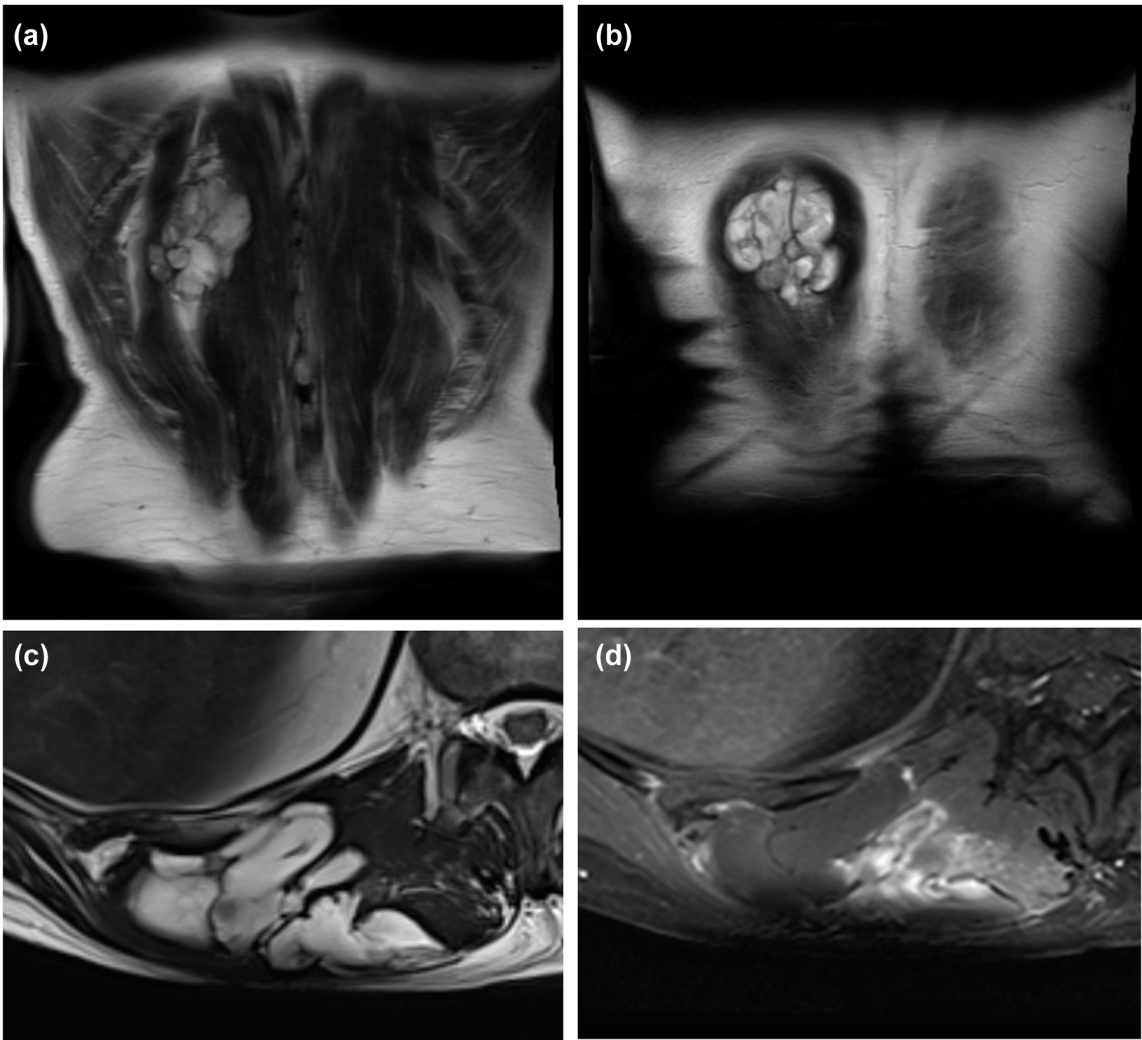
The MRI image of a contrast-enhanced coronal view of myxofibrosarcoma: (a, b) a soft tissue mass adhering to the skin and (c, d) a nodular cystic lesion depicting the size of the tumor with a large myxoid component. MRI-magnetic resonance imaging.

In February 2017, a 55-year-old Polish man was referred to the Oncology Clinic after having a sarcoma resection R1 of the right lumbar region without a previous biopsy. The patient with recurrence of STS of the back, along with part of the paraspinal muscles on the right side, had no prior treatment. MRI examination of the soft tissues of the thoracolumbar region on the right side, at the Th10-L1 level, showed a nodular cystic lesion (with a large myxoid component), the lesion covers the longissimus muscle of the thorax and the iliocostal mass of the lumbar (Figures 3 and 4). The tumor dimensions in the transverse plane are 90 × 40 mm and cc 93 mm.

**Figure 5.**
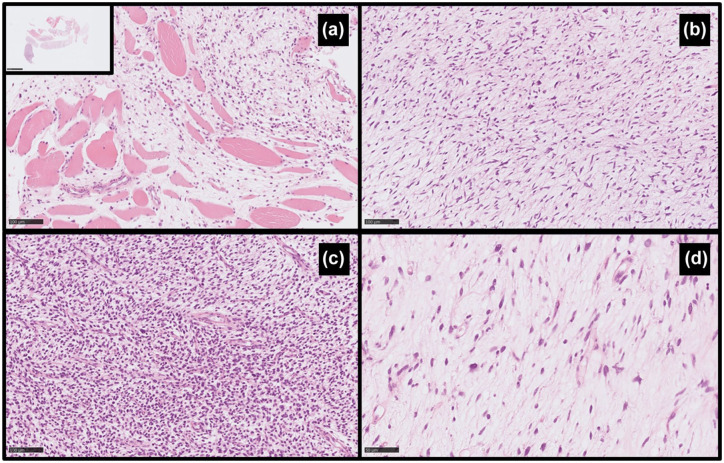
Histopathological image of the hematoxylin and eosin staining of myxofibrosarcoma. (a) Infiltrative type of growth, myxofibrosarcoma between skeletal muscles (200×). (b) Myxoid fields of the tumor with abundant stroma (200×). (c) Dense fields of the tumor, highly cellular with increased grade and mitotic activity (200×). (d) Typical fields with pseudolipoblasts (400×).

Pathological consultation showed a myxoid spindle cell tumor with low to medium cellularity and a medium degree of cytological atypia. Mitotic activity was up to 22/1.734 mm^2^. There was no necrosis. In the stroma, there were numerous arcuate blood vessels. In single cells, positive stainings were observed for CD34, SMA (focally), and caldesmon. Negative stainings were observed for desmin, S100, SOX10, and CK PAN (clone: AE1/AE3). The microscopic image, together with the immunohistochemical profile, suggested a myxoid fibrosarcoma with a higher degree of histological grade (myxofibrosarcoma, G2; Figure 5).

This patient was also enrolled for a phase II UNRESARC clinical trial conducted in our department.^31^ The patient was treated with neoadjuvant chemoradiotherapy. The patient was treated with AI chemotherapy-ifosfamide (2500 mg/m^2^ on days 1–4) and doxorubicin (25 mg/m^2^ on days 1–3). The patient received three prescribed therapy courses between March 31, 2017 and May 15, 2017. Within the week after the first AI infusion, hypofractionated radiotherapy (a total dose of 25 Gy in 5 Gy/fraction) was started on April 5, 2017. The tumor size decreased from 93 mm before treatment to 91 mm after treatment. Tumor resection was performed on June 9, 2017.

After radical resection (resection R0), histopathological examination revealed a subfascial tumor in the pT2 stage with the largest dimension of 65 mm. There were extensive areas of hyalinization within the tumor; preserved tumor tissue stained with H&E after therapy constituted approximately 10% of the tumor microscopically examined. The pathological response score was a D grade according to the EORTC-STBSG recommendations.^33^

The patient was disease-free for over 50 months after treatment. In September 2021, the CT scan showed new lesions and the progression of sarcoma in the lungs. The patient was treated with ADIC chemotherapy between October 2021 and June 2022. The patient received the first three treatment cycles at the dosage—doxorubicin (12–15 mg/m^2^ on days 1–5) and dacarbazine (150–200 mg/m^2^ on days 1–5). In January 2022, a CT scan showed reduced meta changes. After three cycles of treatment, the patient received five more cycles of treatment at a dose reduced by 25%–30% as a result of poor tolerance to treatment. The next CT scan in May 2022 showed further reduced meta lesions. In August 2022, a CT scan showed stabilization of the disease. After 7 months, in March 2023, a CT scan revealed an oligoprogression of the lung lesion. In consequence, the patient received stereotactic radiotherapy (a total dose of 50 Gy/80% in 10 Gy/80% fractions) to the left lung.

However, in June 2023, the next CT scan showed an increase in meta changes. Due to the further progression of the disease, in March 2024, the patient started treatment with gemcitabine–docetaxel chemotherapy at a dose of gemcitabine (625 mg/m^2^, on days 1 and 8) and docetaxel (50 mg/m^2^, on day 8). In July 2024, due to poor tolerance to treatment, the doses were reduced—gemcitabine (800 mg on days 1 and 8) and docetaxel (80 mg on day 8), the target dose regardless of body surface area. In November 2024, the patient was hospitalized for sepsis, influenza virus infection, and pneumonia. Because of this fact, the treatment was terminated. On January 7, 2025, a control CT scan showed no progression of target lesions. However, due to past complications, a break in previous systemic treatment was recommended.

## Laboratory workflow

DNA and RNA were extracted from formalin-fixed paraffin-embedded tissue preparation using an Agencourt FormaPure kit (Beckman Coulter, Brea, CA, USA). For next-generation sequencing (NGS), sequencing libraries were prepared using the hybridization-capture-based TruSight™ Oncology 500 panel (Illumina, San Diego, CA, USA) and sequenced on the Illumina NextSeq 500 platform. The genetic panel assesses 523 cancer-related genes at the DNA level (single-nucleotide variant (SNV), small deletion/insertion, copy number variation) and 55 genes at the RNA level (gene fusions and splice variants). The alignment and small variant calling were performed by PierianDx software. An additional bioinformatic pipeline analyzed the raw data from sequencing. First, the BCL files were converted to FASTQ files using the bcl2fastq software (Illumina). Alignment of reads in the FASTQ files to the GRCh37 reference sequence and gene fusion detection were performed using the Arriba workflow.^34^ This analysis provided data such as the type of translocation, breakpoints, or reading frame, and revealed the presence of two isoforms of a novel *PRUNE2::NTRK2* gene fusion in both cases.

As a confirmation, we used previously extracted RNA and prepared a cDNA library with FusionPlex^®^ Sarcoma v2 (Archer™, Boulder, CO, USA). This NGS panel identifies known and novel fusions, splice variants, SNVs, insertions, deletions, and relative expression with the targeted NGS of 63 genes relevant to sarcoma. The prepared libraries were sequenced on an Illumina NextSeq 500 system. The FASTQ files with the sequencing reads were analyzed using the bioinformatic pipeline described above.

**Figure 6.**
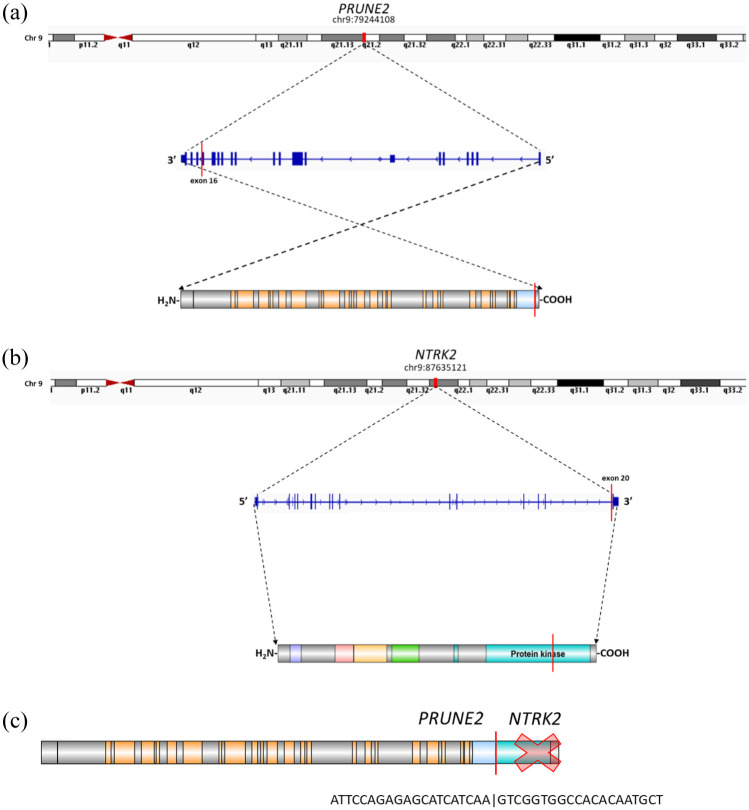
The *PRUNE2::NTRK2* gene fusion scheme—the first isoform. (a) Illustration of the breakpoints in the *PRUNE2* transcript on chromosome 9. (b) Illustration of the breakpoints in the *NTRK2* transcript on chromosome 9. (c) Visualization of the *PRUNE2::NTRK2* gene fusion product with a STOP codon in the *NTRK2* gene sequence.

Each analysis confirmed the presence of two isoforms of *PRUNE2::NTRK2* gene fusion in both cases, with genomic breakpoints of hg19/GRCh37 chr9:79244108 with chr9:87635121 and chr9:79239939 with chr9:87635121. The consensus sequence that was uniquely mapped to *PRUNE2* and *NTRK2* was analyzed using BLAT (the BLAST-like alignment tool),^35^ and split-read sequences were identified using Integrative Genomics Viewer. Both genes encode numerous alternatively spliced transcripts; therefore, the Ensembl GRCh37 was used for the identification of specific transcripts. In the first gene fusion isoform, the *PRUNE2* (NM_015225, ENST00000376718) breakpoint was located at the end of exon 16 (chr9:79244108) on the reverse strand (Figure 6(a)), whereas the *NTRK2* (NM_006180, ENST00000376214) breakpoint was located at the start of exon 20 (chr9:87635121) on the forward strand (Figure 6(b)). Both isoforms of the gene fusion product contain a sequence upstream of the breakpoint of the *PRUNE2* gene and a sequence downstream of the breakpoint of the *NTRK2* gene. Both gene fusion isoforms, however, contain a frameshift that affects the sequence of the TrkB domain and creates a STOP codon at the end of exon 20 of the *NTRK2* gene (Figure 6(c)).

**Figure 7.**
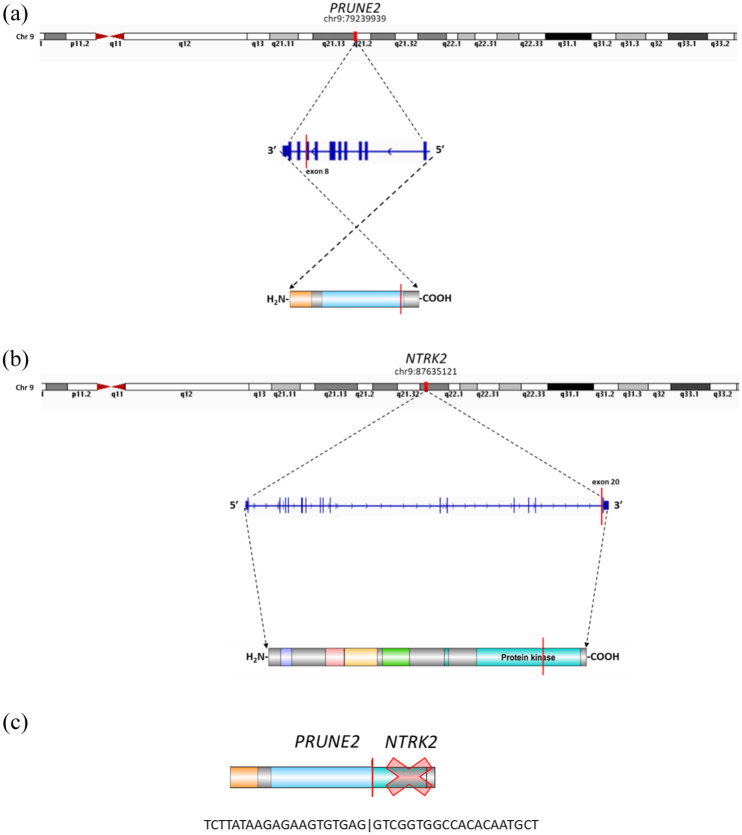
The *PRUNE2::NTRK2* gene fusion scheme—the second isoform. (a) Illustration of the breakpoints in the *PRUNE2* transcript on chromosome 9. (b) Illustration of the breakpoints in the *NTRK2* transcript on chromosome 9. (c) Visualization of *PRUNE2::NTRK2* gene fusion product with STOP codon in the *NTRK2* gene sequence.

In the second gene fusion isoform, the *PRUNE2* (ENST00000424866) breakpoint was located at the end of exon 8 (chr9:79239939) on the reverse strand (Figure 7(a)), while the *NTRK2* (ENST00000376214) breakpoint was located at the start of exon 20 (chr9:87635121) on the forward strand (Figure 7(b)). This gene fusion isoform contains a shorter version of the *PRUNE2* gene and again is out-of-frame *NTRK2* gene (Figure 7(c)).

The reporting of this study conforms to the CARE guidelines (see the Supplemental Material Files).

## Discussion

This study described two cases in which a novel fusion of *PRUNE2::NTRK2* genes was identified in two adult STS patients. The *PRUNE2* gene encodes a 340 kDa protein that contains a conserved scaffold domain of Bcl2−/adenovirus E1B 19 kDa-interacting protein 2 (BNIP2) and Cdc42GAP homology (BCH) at its C-terminus.^27,29^ This gene is also named the *BMCC1* gene (for BCH motif-containing molecule at the carboxyl terminal region 1).^36^ BCH domains directly bind to small GTPases and their regulators—GTPase-activating proteins and guanine nucleotide exchange factors to modulate signaling cascades such as MAPK and Rho signaling and apoptosis.^37^ The PRUNE2 protein harbors a DHH motif (His-Asp-As) at its N-terminus—the PRUNE1 homology region.^36^ The DHH motif has cytoplasmic cyclic nucleotide phosphodiesterase (PDE) activity and chelates Mg^2+^ ions, which is crucial for PDE activity and may have an impact on cell motility and apoptosis.^38,39^ PRUNE2 belongs to the Bcl-2/adenovirus E1B 19 kDa-interacting proteins (BNIPs) family involved in cellular processes such as morphogenesis, differentiation, motility, and apoptosis through interactions with signaling networks.^27,29^ Increased PRUNE2 expression is correlated with a favorable prognosis in neuroblastoma and prostate cancer; its association with survival in leiomyosarcoma patients is unclear. However, elevated protein expression indicates a positive prognosis in leiomyosarcoma.^28,29^

In the first gene fusion isoform, the *PRUNE2* (ENST00000376718) breakpoint was located in exon 16 at the end of the sequence encoding the BCH domain, leading to substitutions of the last six amino acids in this domain. It is not known whether these substitutions change protein function. However, the most important amino acids for the catalytic function of the BCH domain did not change. The amino acid sequences of the PRUNE2 BCH domain homologous to the BCH domain family of proteins, such as BNIP-2, which are essential for the BCH domain function, are conserved. The altered amino acids from the C-terminus of the protein differ between the homologous proteins.^40^ This suggests that the PRUNE2 BCH domain may still function, despite the change in the last six amino acids. However, without additional studies, it is not possible to confirm whether the change in the last amino acids will affect the function of the BCH domain and the tumor suppressor function of the PRUNE2 protein.

In the second gene fusion isoform, the *PRUNE2* (ENST00000424866) breakpoint was located in exon 8, also leading to substitutions of the last six amino acids in the BCH domain, like in the previously described case. However, it is not known whether this shorter isoform of the PRUNE2 protein is functional. This isoform in the Ensembl GRCh37 and the SwissProt/UniProt^41^ database contains 260 amino acids, in contrast to the first PRUNE2 isoform, which contains 3088 amino acids. This shorter isoform does not contain the DHH motif at its N-terminus. Therefore, the activity of this isoform is unknown. Homologous proteins containing the BCH domain lack the DHH motif.^40^ This suggests that the lack of the DHH motif should not significantly affect the function of the BCH domain itself, but the functions of this isoform of PRUNE2 may be modified or limited relative to the native version of PRUNE2 protein.

The *NTRK2* oncogene plays a role in proliferation by initiating the MAPK signaling pathway, which promotes tumor progression.^16^ *NTRK*-associated rearrangements are significant in various cancers, including lung, colorectal, thyroid, central nervous system cancers, melanoma, and hematologic neoplasms.^16,26^ These fusions are important biomarkers and have prognostic implications, particularly in lung squamous cell carcinoma and neuroblastomas.^16^ Preclinical studies with mouse models of genetically engineered *NTRK*-associated gene fusion cancers have been shown to develop highly aggressive tumors.^17^ Furthermore, Bazhenova et al.^42^ showed that in various types of cancer, the overall survival (OS) of patients with *NTRK-*associated fusions could be reduced. STS patients had a 5-year OS rate of 78% in 2013–2019.^43^ However, the 2-, and 5-year OS rates of STS with metastatic disease were 49.9% and 24.8%, respectively.^18,44^ Median OS were between 12 and 24 months.^44–46^ STS patients treated in the clinical trial UNRESARC (NCT03651375) conducted in our department were estimated to have 2- and 3-year OS rates of 67% and 53%, respectively.^31^ However, both patients with detected *PRUNE2::NTRK2* gene fusion are currently alive, more than 7 years after starting treatment at our sarcoma center. In the case of *PRUNE2::NTRK2* gene fusion described here, both gene fusion isoforms contain a frameshift that affects the sequence of the TrkB domain and creates a STOP codon, resulting in synthesis of a different amino acid sequence and loss-of-function of TrkB. Such an atypical *NTRK*-associated gene fusion will possibly contribute to a lower aggressive potential of the tumor, compared with the typical cases that result in elevated Trk activity.

The discovery of *NTRK*-associated gene fusions has led to the development of FDA-approved targeted therapies, for example, larotrectinib and entrectinib, which have shown efficacy in multiple cancer types by providing personalized treatment options.^16,26,47^ However, in Poland, Trk inhibitor treatment has been reimbursed only since July 1, 2023. To obtain targeted treatment, patients must fulfill certain criteria, including receiving all available treatment regimens according to National Comprehensive Cancer Network recommendations. Our patients received standard treatment with good outcomes and stabilization of the disease. Due to their current health condition, they did not receive targeted treatment with Trk inhibitors in the next line of treatment. Moreover, amino acid substitutions in the Trk kinase domain can result in resistance to Trk inhibitors (larotrectinib and entrectinib), causing steric hindrance to inhibitor binding, inducing conformational changes in the Trk kinase domain, or altering the affinity for ATP.^48–50^ The *PRUNE2::NTRK2* gene fusion detected in our patients results in an out-of-frame *NTRK2* gene. This alteration causes the synthesis of a different amino acid sequence and a lack of the TrkB kinase domain, suggesting possible resistance to Trk inhibitors (larotrectinib and entrectinib).

On the other hand, the *NTRK2* breakpoint was located in the sequence of the kinase domain. Nevertheless, the MAPK protein-interacting region is located between 455 and 466 amino acids in the SwissProt/UniProt^41^ database upstream of the kinase domain. Therefore, besides the gene fusion, the *NTRK2* gene sequence should encode a shorter version of the TrkB protein without the kinase domain. Synthesis of the TrkB protein with a functional MAPK protein-interacting region and without the kinase domain may lead to a dominant-negative role of the receptor, which, in consequence, inhibits the TrkB signaling.^51^ The dominant-negative receptor may enhance the potential suppressor role of the *PRUNE2::NTRK2* gene fusion. However, our analysis did not include the *NTRK2* sequence beyond the fusion; therefore, we do not know whether alternative splicing would provide the sequence modifications.

Studies suggest that oncogene-related fusions tend to be overexpressed, while fusions involving tumor suppressor genes show low expression.^52^ The *PRUNE2::NTRK2* gene fusion consists of a tumor suppressor gene and an oncogene. However, in both isoforms of this gene fusion, the out-of-frame *NTRK2* gene with a STOP codon provides loss-of-function TrkB. PRUNE2 has a tumor suppressor function, but the function of PRUNE2 in the fusion protein is unknown. The two isoforms of *PRUNE2::NTRK2* gene fusions identified in our study may, however, lead to modified or limited tumor suppressor function of PRUNE2 or even loss-of-function and consequently, a possible dominant-negative effect. Properly understanding the molecular impact of *PRUNE2::NTRK2* gene fusion will require further investigation involving a larger group of patients and assessment of native protein expression levels in patients without the gene fusion.

## Conclusion

In conclusion, we report a novel fusion of the *PRUNE2::NTRK2* genes in STS. This study highlights the importance of genetic profiling in STS for personalized cancer treatment, which may improve patient outcomes. However, genetic profiles should be evaluated by an experienced genetic specialist to confirm the need for a treatment order. In this study, we showed the *PRUNE2::NTRK2* gene fusion with a potential suppressor effect due to the functions of the PRUNE2 protein and the non-functional TrkB protein. Furthermore, the novel gene fusion may provide a dominant-negative function of TrkB, which may enhance the tumor suppressor role of this gene fusion. The PRUNE2 function in the described gene fusion could, however, also be opposite if the amino acid substitutions in the BCH domain sequence affect protein function. The clinical impact of the *PRUNE2::NTRK2* gene fusion is still unknown and needs to be investigated.

## Supplemental Material

sj-pdf-1-tam-10.1177_17588359251395379 – Supplemental material for Identification of a novel PRUNE2::NTRK2 gene fusion in soft tissue sarcoma patients—friend or foe? Case series Supplemental material, sj-pdf-1-tam-10.1177_17588359251395379 for Identification of a novel PRUNE2::NTRK2 gene fusion in soft tissue sarcoma patients—friend or foe? Case series by Klaudia Bobak, Andrzej Tysarowski, Katarzyna A. Seliga, Jakub Pia̧tkowski, Paweł Golik, Mateusz J. Spałek, Anna Szumera-Ciećkiewicz, Piotr Rutkowski and Anna M. Czarnecka in Therapeutic Advances in Medical Oncology

## Declarations

**Ethics approval and consent to participate:** The present study was approved by the Ethics Committee of the Maria Sklodowska-Curie National Research Institute of Oncology (approval number: 8/2017/1/2018) on May 17, 2018. Written informed consent was obtained from all participants in a phase II clinical trial UNRESARC (NCT03651375). Consent to participate: Written informed consent to use histopathological samples and pathological diagnostic reports for research purposes was obtained from all patients prior to surgery. They were given the option to refuse to participate by opting out.

**Consent for publication:** One patient (case 2) provided his written informed consent for publication, and the other, due to the patient’s health condition, provided verbal consent for publication (case 1).

**Availability of data and materials:** Not applicable.
